# Effect of Soil Composition on Secondary Metabolites of Moroccan Saffron (*Crocus sativus* L.)

**DOI:** 10.3390/plants12040711

**Published:** 2023-02-06

**Authors:** Soukaina Chaouqi, Natalia Moratalla-López, Gonzalo L. Alonso, Cándida Lorenzo, Abdelmjid Zouahri, Nazha Asserar, El Mehdi Haidar, Taoufiq Guedira

**Affiliations:** 1 Laboratory of Organic Chemistry, Catalysis and Environment, Faculty of Sciences, Ibn Tofail University, BP 242, Kenitra 14000, Morocco; 2 Cátedra de Química Agrícola, ETSI Agrónomos y de Montes de Albacete, Universidad de Castilla-La Mancha, Campus Universitario, 02071 Albacete, Spain; 3 Environment and Natural Resources Conservation Research Unit, INRA, CRRA, BP 6356, Rabat 10000, Morocco; 4 Laboratory of Botany and Plant Protection, Faculty of Sciences, Ibn Tofail University, BP 133, Kenitra 14000, Morocco; 5 Department of Mineral Chemistry, Mining Laboratories Division, ONHYM, Campus: 34, Avenue Al Fadila, City Yakoun El Mansour, BP 8030, Rabat 10000, Morocco

**Keywords:** spice quality, crocins, safranal, picrocrocin, mineral elements, soil texture, HPLC-DAD, ED-XRF fluorescence, X-ray diffraction

## Abstract

Climate and soil are important factors that affect the quality of saffron. Saffron quality is determined by the marked content of secondary metabolites. The objective of this work was to study the effect of soil physicochemical properties on the secondary metabolites of saffron. Our study concerned the analysis of saffron samples by high-performance liquid chromatography-detection by diode array (HPLC-DAD). Soil samples were analyzed by physicochemical methods, ED-XRF fluorescence and X-ray diffraction to determine the different types of clays. Saffron samples grown in loam–clay–sand soils contained high values of crocins and kaempferol 3-sophoroside 7-glucoside but low values of safranal. In addition, saffron samples grown in soils rich in organic matter, phosphorus and potassium contained high values of crocins and kaempferol 3-sophoroside 7-glucoside but low values of safranal. This original approach was carried out for the first time in our study, both by ED-XRF fluorescence and by X-ray diffraction, to determine what elements affect the quality of saffron. Thus, we concluded that clays containing low amounts of iron could have a positive effect on the coloring strength of saffron.

## 1. Introduction

As saffron is a medicinal plant, it is important not only to obtain the best yield but also to obtain high content of secondary metabolites in order to have the best quality of saffron. Quality control is important due to consumer and regulatory requirements [1]. Crocins, picrocrocin and safranal are the main secondary metabolites that determine the quality of saffron. Many factors can influence the retention and formation of these metabolites, such as environmental conditions [2,3,4,5,6,7], agricultural practices [8,9], corms [1,10,11], harvest period [12] and drying and storage methods [13,14,15,16,17,18]. These factors have a direct or indirect effect on the accumulation of secondary metabolites [2,8,19,20].

Regarding environmental conditions, soil is the most important factor stimulating secondary metabolites, as it controls the movement and availability of air, nutrients and water. Atyane et al. (2017) [21] showed that the chemical composition of saffron is influenced by geographical origin, soil and climate. In addition, Mykhailenko et al. (2020) [22] reported that soil type, altitude, temperature, irrigation cycles and harvesting periods affect the accumulation of compounds in saffron plants used as medicinal and pharmaceutical raw materials and food additives. Conflicting results were obtained by Lambert and Karra (2016) [23] and El Grah et al. (2022) [24]. Both groups of researchers have shown that neither soil type nor irrigation water have an effect on the chemical quality of saffron.

According to the researchers’ results, the accumulation of secondary metabolites is strongly dependent on various environmental factors, and for most plants, a change in an individual factor can alter the content of secondary metabolites even if other factors remain constant. The morphological adaptability of plants to the environment is relatively easy to observe and recognize, while the biochemical adaptability of plants is relatively difficult to observe. The synthesis of some natural products can be altered by various abiotic factors. Notably, individual environmental stress can selectively increase the content of several secondary metabolites in plants [25].

In this study, we have chosen to work on an individual factor: the soil. This study contributes to evaluating the influence of the physicochemical properties of soil on saffron secondary metabolites in order to determine the best soil to guarantee the best quality of saffron in the Taouyalt region. For this, we used ED-XRF fluorescence and X-ray diffraction to analyze other important elements present in soil, in addition to those analyzed by physicochemical techniques, which could affect the quality of saffron.

## 2. Results and Discussion

### 2.1. Effect of Soil Composition on Secondary Metabolites of Saffron

**Figure A1:**
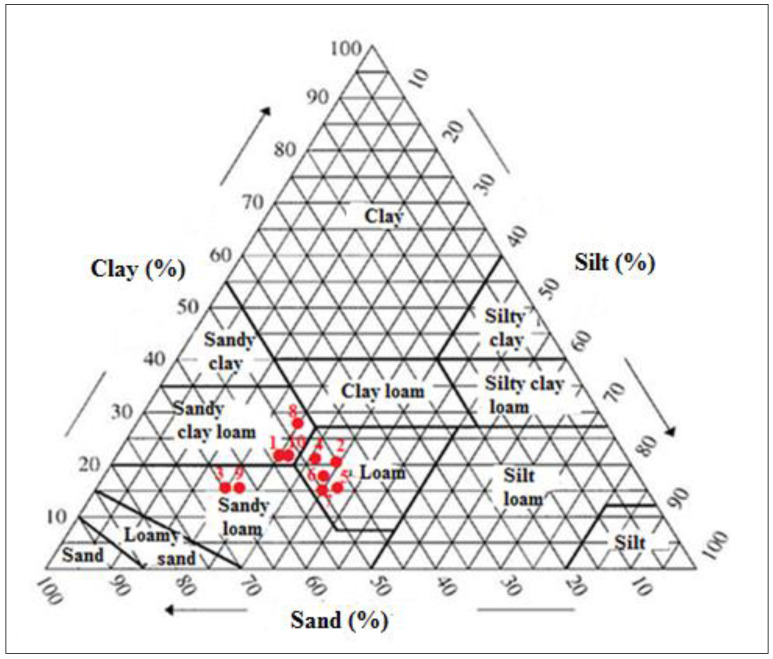
Triangular diagram of textural classes of the studied soils.

The chosen parameters were analyzed separately to determine those responsible for the variation of secondary compounds in saffron. According to Tukey’s test, the mean values of all secondary metabolites vary significantly from one site to another. Table 1 presents the results of the physicochemical analysis of the soil samples. Table 2 and Table 3 present the effect of texture, pH, mineral elements and organic matter of soil on the main secondary metabolites of saffron. Figure A1 presents the USDA texture triangle [26], which classifies soil samples according to the percentages of clay, loam and sand.

#### 2.1.1. Influence of Soil Texture

Analysis of variance showed that soil texture had a significant effect on crocins, safranal and kaempferol 3-sophoroside 7-glucoside. We found that saffron grown in loam–clay–sand soils contained higher values of crocins and kaempferol 3-sophoroside 7-glucoside and lower values of safranal compared to that grown in loamy or sand–loam soils. In addition, we found a positive correlation between clay and kaempferol 3-sophoroside 7-glucoside and a positive correlation between clay and *trans*-3-Gg.

The results we obtained are not consistent with those obtained by Birouk et al. (2011) [27]; they found a positive correlation between clay and the aromatic strength of saffron. It should be noted that Birouk et al. (2011) [27] worked on saffron samples from different environments, which could have influenced the results. Our results are in agreement with those obtained by Cardone et al. (2020) [28], who showed that the concentrations of crocins and picrocrocin are low in sandy and sand–loam soils. The low values of secondary metabolites in sandy and sand–loam soils are explained by the more flexible structure of these soils, which gives less resistance to root growth. Thus, it reduces the coloring strength and production of compounds responsible for the bitter taste associated with stress [29]. Furthermore, Husaini et al. (2010) [30] suggested that increased clay content has a significant impact on soil water supply, nutrient absorption and thus, saffron quality, especially safranal content. On the other hand, El Grah et al. (2022) [24] showed that the chemical quality of saffron is not affected by the type of soil, knowing that the clay content in the analyzed samples was less than 10%.

#### 2.1.2. Influence of Soil pH

Slightly higher values of *cis*-3-Gg were observed in saffron samples grown in soils with a moderately alkaline pH compared to those grown in soils with a neutral or weakly alkaline pH.

#### 2.1.3. Influence of Soil Phosphorus

Phosphorus has a significant effect on kaempferol 3-sophoroside 7-glucoside and crocins, particularly, *cis*-4-GG, *trans*-2-G and *cis*-3-Gg. Saffron samples grown in soils with high phosphorus content contained higher values of *cis*-4-GG, *trans*-2-G, *cis*-3-Gg and kaempferol 3-sophoroside 7-glucoside compared to those grown in soils rich or low in phosphorus. On the other hand, slightly high concentrations of safranal were observed in soils with low phosphorus content.

#### 2.1.4. Influence of Soil Potassium

Analysis of variance showed that potassium had a significant effect on crocins, kaempferol 3-sophoroside 7-glucoside and picrocrocin. The values of *trans*-4-GG, *trans*-2-gg, *cis*-3-Gg, *trans*-1-g, kaempferol 3-sophoroside 7-glucoside and picrocrocin were the highest in saffron samples grown in high- to very-high-potassium soils. In contrast, growing *Crocus sativus* in soils with low potassium content produced saffron with the highest safranal concentration. These results are in agreement with those obtained by Yarami and Sepaskhah (2016) [31], who showed that secondary metabolites are positively correlated with phosphorus and potassium.

#### 2.1.5. Influence of Soil Organic Matter

Several studies have shown that organic matter improves the biosynthesis of secondary metabolites in many aromatic and medicinal plants [32,33]. According to Tukey’s test, organic matter has a significant effect on safranal, coloring strength and crocins, particularly *trans*-4-GG. We observed that saffron samples grown in soils very rich in organic matter contained the highest concentrations of *trans*-4-GG but the lowest concentrations of safranal. This can be explained by the fact that the increase in the nutrient availability of essential elements and that of the cation exchange capacity (CEC) could promote the biosynthesis of crocins and their accumulation in the styles/stigmas [34]. This result is in agreement with that obtained by Rezaian and Paseban, (2006) [35], who reported that crocin and picrocrocin contents increase with the application of cattle manure, while safranal concentration decreases. In addition, Rabani-Foroutagheh et al. (2013) [36] reported that foliar fertilization, characterized by high nitrogen and phosphorus contents, improves saffron quality, showing a positive effect on crocin but a negative effect on safranal. On the other hand, Ghanbaria et al. (2019) [37] showed that organic amendments and chemical fertilizers positively affect the aroma and flavor of saffron. Moreover, chemical fertilizers have a significant influence on safranal and picrocrocin content. Ghanbaria et al. (2019) [37] also showed that the total phenol content and the antioxidant activity of saffron stigmas improves with organic amendments.

According to our results, among soil properties, texture seems to be the main factor stimulating secondary metabolites, as it controls the movement and availability of air, nutrients and water. Thus, we carried out an analysis by X-ray diffraction to determine the types of clays that constitute the studied soils. X-ray fluorescence analysis was performed to assess the effect of other elements present in soils, which are SiO_2_, Al_2_O_3_, CaO, Fe_2_O_3_, K_2_O, MgO, MnO, Na_2_O, P_2_O_5_ and TiO_2_.

The mineralogical composition of the studied soils is characterized by the presence of quartz and clays, which include aerinite, albite, muscovite and illite. Table 4 shows the chemical formulas of the identified clays.

**Figure A2:**
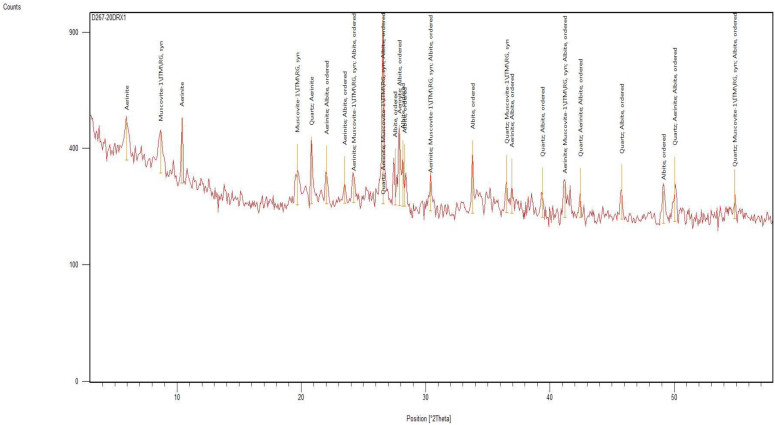
Diffractogram of X-rays on powder of the T1 sample.

Figure A2 shows an X-ray powder diffractogram of a soil sample (T1) with the clays identified. Due to the sheet structure, several families of clays have sheets of identical thickness. An analysis by X-ray diffraction on oriented blades was performed. This enabled the orientation of the clay particles according to the (001) plane. To reinforce the reflections of the family of these plans, treatments enabled the evolution of clays to be followed precisely:

- Ethylene glycol treatment: the blade is exposed to ethylene glycol vapor for 2 h to induce the swelling of some types of minerals.
- Heating: this treatment heats the blade for 2 h at 550 °C.

By comparing the diffractograms, we could identify the clays present in each sample.

**Figure A3:**
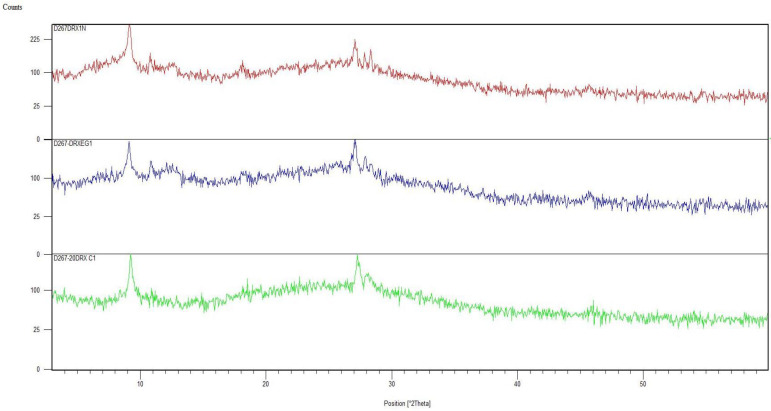
Diffractogram of X-rays on oriented blades of the T1 sample.

Figure A3 shows the X-ray diffractogram of the oriented blades of the same sample (T1), which confirmed the identifications obtained for the clays.

The results showed that quartz (SiO_2_) was the most abundant compound in the studied soil samples. Saffron samples grown in quartz-rich soils had the highest values of coloring strength. These soils contained less iron and manganese and high potassium compared to the other soil samples (Table 5). The decrease in iron and manganese in these soils can be explained by the ability of silicon to regulate these elements, limiting their risk of toxicity. In addition, silicon plays an important role in plant defense against abiotic stresses, including metal toxicity, drought, salinity and nutrient imbalances, and biotic stresses, including insects and pathogens [38,39]. There is now ample evidence that the application of silicon in soil leads to greater silicon accumulation in plant tissues than foliar application does [38,40].

Rogalla and Römheld (2002) [41] and Liang et al. (2007) [42] showed that the toxicity of manganese is reduced in plants fertilized with silicon because it increases the bond of manganese with the cell walls, thus limiting its cytoplasmic concentration. Moreover, silicon leads to a homogeneous distribution of manganese in the leaves, which limits the formation of localized necrosis [43,44].

Studies on rice suggest that silicon application increases the ability of roots to oxidize iron, converting ferrous ion (Fe^2+^) to ferric ion (Fe^3+^). This oxidation reduces iron absorption, which reduces its toxicity [45].

The high potassium values in the soil samples are explained by the presence of muscovite and illite. Barre et al. (2006) [46] showed that the content of clay mineral potassium is strongly related to the amount of illite layers. They assume that illite has a low ability to bind and release exchangeable ions such as calcium, magnesium and sodium but has a high potential for potassium accumulation. Illite is the soil mineral richest in potassium. Indeed, intensive agricultural practices without potassium fertilization reduce illite content in surface soils, while several years of potassium fertilization without plant growth can increase illite content. On the other hand, we observed that dark soils produce saffron with degraded quality compared to that of light soils. Red, orange, yellow, green and blue colors in soil (clay or not) are due to the state of iron in the soil (Fe^3+^ in the former three cases and Fe^2+^ in the latter two). When the soil has a color that tends towards white, it means that this element has been dissolved. The deterioration in the quality of saffron, particularly its coloring strength, can be explained by an excess of iron in the soil. Although iron is not used in the synthesis of carotenoids, it remains essential for their formation [47]. We noticed that the coloring strength of saffron is high in soils rich in silica with high concentrations of phosphorus. This effect is attributed to the decreased absorption of iron and manganese under high phosphorus levels [48]. In fact, these soils have the lowest iron content. This may confirm that low doses of iron could have a positive effect on the coloring strength of saffron.

Farahani et al. (2015) [49] studied the effect of iron-chelated nanofertilizers on the quantitative and qualitative characteristics of saffron. They found that this fertilizer is more effective than conventional iron-chelated fertilizers. They recommend using this fertilizer and minimizing the use of other common fertilizers in saffron production. Through the use of nanofertilizers as an alternative to traditional iron-chelating fertilizers, iron is released gradually and in a controlled manner, thus providing nutrients to plants more efficiently [50].

The use of nanotechnology, especially nanofertilizers, to precisely control the release of nutrients can be an effective step towards sustainable and environmentally friendly agriculture [51,52]. It can also be an effective step towards increasing the quantity and quality of saffron.

Miao et al. (2010) [53] showed that the addition of sodium silicate increases the concentration of potassium in the leaves, stems and roots of potassium-deficient soybean plants. Silicon also reduces the foliar gas exchange parameters and hydraulic conductance of the whole plant. In addition, it increases potassium accumulation in the xylem of potassium-deficient sorghum plants. This indicates that silicon improves the water status of plants under potassium deficiency [54]. Mali and Aery (2008) [55] observed that, even at low silicon concentrations, there is an increase in potassium absorption by wheat plants.

The second most abundant compound after silica is alumina (Table 5). However, aluminum is necessary only in low doses for plants. Previous studies suggest that silicon and aluminum interact in the soil to create inert aluminosilicate colloids; this reduces phytotoxic concentrations of aluminum in soil solutions [42,56]. Silicon can also promote the production of phenolic exudates from roots that chelate free aluminum, thus reducing its absorption by maize roots [57]. These mechanisms of detoxification are external to the plant. It has also been shown that aluminum can be detoxified by the internal mechanisms of the plant, which form aluminosilicates in the root apoplasm [58,59] or sequestrate aluminum in phytoliths, resulting in reduced aluminum toxicity in the aerial parts [60,61].

An increase in crocins, kaempferol 3-sophoroside 7-glucoside and picrocrocin was observed in loam–clay–sand soils, which are low in calcareous, slightly alkaline and rich in organic matter (T10, T2 and T1). These results are consistent with those obtained by de Cardone et al. (2020) [28], who evaluated the impact of the physical and chemical properties of soil on the growth, yield and quality of saffron (*Crocus sativus* L.). Arno et al. (2012) [62] evaluated the effect of soil and crop nutritional properties on the yield and quality of grapes. They found that soils with a high content of calcium carbonate have limitations in terms of the availability of microelements, which leads to a decrease in phenol content and, therefore, in the color of the grapes.

The main compound affecting saffron aroma is safranal. Safranal is strongly affected by post-harvest processes, especially drying and storage [17,18]. We found that the concentration of safranal was high in soils with a loam–sand or loamy texture and that were slightly calcareous. This may be an adaptation mechanism under conditions unfavorable to crop growth, such as nutrient deficiencies, particularly that of phosphorus. Deficiency of phosphorus may be due to its complexation in the form of alumina phosphates, preventing its migration into the plant. These results are consistent with those reported in the literature. Some authors have shown a significant effect of soil properties on the variation in the composition of essential oils of different medicinal plants [34,63,64,65]. A positive correlation between volatile terpenes and calcium carbonate has also been found in other crops. Ormeno and Fernandez (2012) [66] reported that *Pinus halepensis* Mill., *Cistus halbidus* L. and *Myrtus communis* L. contain higher amounts of terpene in calcareous soils than in siliceous soils. In addition, Mumivand et al. (2011) [67] showed that calcium carbonates increase the concentration of the main compounds of essential oils (carvacrol, *γ*-terpinene and *β*-bisabollene) in *Satureja ortensis* L.

It should be noted that all saffron samples were grown at high altitudes. This could also have an effect on the aroma and coloring strength of the spice. It has been shown that high altitudes and low temperatures have a positive effect on the production of secondary metabolites. The increase in the content of phenolic compounds and carotenoids with increasing altitude is explained as a response to increased UV radiation. It is also assumed that carotenoid biosynthesis is affected by the environment, especially temperature; the amount of carotenoids increases with decreasing temperature. Furthermore, Mykhailenko et al. (2020) [22] showed that increased crocin and picrocrocin concentrations at high altitudes could be due to total precipitation, air temperature, solar radiation and soil characteristics, which significantly affects the accumulation of secondary compounds of saffron. Previous studies indicate that agronomic and climatic factors, particularly altitude, affect the quality of saffron; crocins content increases with increasing altitude [2,7]. Kothari et al. (2021) [7] observed the opposite behavior for safranal; safranal concentration decreases with increasing altitude. They also showed that phenolic compounds and total flavonoids are significantly higher in saffron grown at high altitudes compared to that grown at low altitudes. Additionally, annual precipitation is higher in high-altitude regions than in low-altitude regions [7]. The amount of saffron’s secondary metabolites also depends on corm age, with the youngest saffron samples having the highest concentrations of secondary metabolites [68,69].

## 3. Materials and Methods

### 3.1. Analysis of Saffron Samples

#### 3.1.1. Samples

The experiments were carried out on samples of pure saffron obtained directly from the producers of Taouyalte (rural commune of Taliouine, Morocco). They were dried in the shade and then stored in smoked glass boxes away from light.

#### 3.1.2. Standards

Safranal with purity ≥88% was obtained from Sigma-Aldrich. Crocetin esters: *trans*-crocetin di (*β*-D-gentiobiosyl) ester (*trans*-4-GG) and *trans*-crocetin (*β*-D-glucosyl)-(*β*-D-gentiobiosyl) ester (*trans*-3-Gg) of purity ≥99% were obtained from Phytolab GmbH and Co. KG. Kaempferol 3-sophoroside 7-glucoside with purity ≥97% was obtained from Sigma-Aldrich (Madrid, Spain).

#### 3.1.3. Solvents

Acetonitrile was obtained from Panreac (Barcelona, Spain), and water was purified using a Milli-Q system (Millipore, Bedford, MA, USA).

#### 3.1.4. Preparation of Saffron Extract

Aqueous extracts of saffron were prepared according to the ISO/TS 3632 standards (2011) [70]. Fifty milligrams of powdered saffron were placed in a 100 mL volumetric flask filled with Milli-Q water. The suspension was stirred at 1000 rpm for 1 h away from light. It was filtered through a polytetrafluoroethylene (PTFE) filter with a pore size of 0.45 μm then transferred directly into vials for analysis by HPLC-DAD.

#### 3.1.5. UV–Visible Spectroscopy Analysis

The same extract prepared for HPLC analysis was diluted for analysis by UV–vis spectroscopy. The measurement of $E_{1cm}^{1\%}$ of an aqueous saffron extract at 440 nm was carried out using a 1 cm quartz cell. The results obtained by direct reading of the absorbance D at 440 nm were as follows: coloring strength was determined to be $E_{1cm}^{1\%}$ 440 nm [70].

#### 3.1.6. HPLC-DAD Analysis

This analysis was carried out according to the method described by García-Rodríguez et al. (2014) [71], with some modifications, such as analysis time. Each analysis was carried out in triplicate, and one measurement was taken for each replicate in order to avoid the degradation of some crocins at the time of the analysis. From each prepared extract, 20 μL was injected into an Agilent 1200 HPLC chromatograph (Palo Alto, CA) equipped with a 150 × 4.6 mm inner diameter and a 5 μm Phenomenex (Le PecqCedex, France) Luna C18 column that was equilibrated at 30 °C. The eluents were water (A) and acetonitrile (B) with the following gradients: 20% B, 0–5 min; 20–80% B, 5–15 min; and 80% B, 15–20 min. The flow rate was 0.8 mL/min. The DAD detector (Hewlett-Packard, Waldbronn, Germany) was set at 250, 330 and 440 nm for the detection of picrocrocin, safranal and crocins, respectively.

*Trans*-4-GG and *trans*-3-Gg were identified using their UV–vis spectrum and retention time using the HPLC-DAD method at 440 nm and with the parameter %III/II of their standards [72]. Picrocrocin and safranal were identified by combining the UV–vis spectrum and retention time using HPLC-DAD at 250 nm and 330 nm, respectively.

The quantification of *trans*-4-GG, *trans*-3-Gg, picrocrocin and safranal was based on calibration curves that were made in the study of García-Rodríguez et al. (2014) [71] as follows:

The calibration curve of *trans*-4-GG concentration, d (mg/L), as a function of its HPLC peak area, b, showed good linear regression in the range from 0.80 to 50.00 mg/L with the equation d = 0.0075b − 0.008 and R^2^ = 0.999. The calibration curve of *trans*-3-Gg concentration, f (mg/L), as a function of its HPLC peak area, g, showed good linear regression in the range from 0.80 to 25.00 mg/L with the equation f = 0.0071g − 0.0047 and R^2^ = 0.999. The calibration curve of picrocrocin concentration, j (mg/L), as a function of its HPLC peak area, k, showed good linear regression in the range from 2.00 to 315.00 mg/L with the equation j = 0.0029k + 0.5194 and R^2^ = 0.999. The calibration curve of safranal concentration, h (mg/L), as a function of its HPLC peak area, i, showed good linear regression in the range from 0.03 to 4.00 mg/L with the equation h = 0.0323i + 0.051 and R^2^ =0.998.

Isomeric standards for *cis*-crocetin esters were identified using the UV–vis spectrum. Molecular coefficient absorbance values for *trans*-4-GG and *trans*-3-Gg in 50% acetonitrile/water (*v*/*v*) were 106,922.75 and 92,781.76 M^−1^ cm^−1^ (440 nm), respectively [71], which are close to the values obtained by other authors for *trans*-4-GG: 89,000 M^−1^ cm^−1^, 132,200 M^−1^ cm^−1^, 133,750 M^−1^ cm^−1^ [73] and 133,500 M^−1^ cm^−1^ [74]. These differences may be due to the use of different solvents. The quantification of *cis* isomers was based on the following equation:

C*_cis_* = (m*_trans_* × ε*_cis_*/ε*_trans_*) area*_cis_* + (n*_trans_* × ε*_cis_*/ε*_trans_*)

where C*_cis_* is the concentration of *cis*-crocetin esters in mg/L, m*_trans_* is the slope of the *trans*-crocetin ester, ε*_cis_*/ε*_trans_* is the *cis*/*trans* ratio obtained by Speranza et al. (1984) [75], area*_cis_* is the peak area of *cis*-crocetin esters and n*_trans_* is the intercept of the *trans*-crocetin ester.

The identification of kaempferol 3-sophoroside 7-glucoside was carried out according to the method described by Chaouqi et al. (2018) [18] by combining its UV–vis spectrum and its retention time using HPLC-DAD at 330 nm. Its quantification was based on the calibration curve. To do this, a series of standard solutions of kaempferol 3-sophoroside 7-glucoside was prepared in a 20% acetonitrile/water (*v*/*v*) with concentrations of 125.00, 62.50, 37.50, 18.75, 7.50, 1.50 and 0.75 mg/L and then analyzed in duplicate by HPLC-DAD. Therefore, the calibration curve of kaempferol 3-sophoroside 7-glucoside concentration, a (mg/L), as a function of its HPLC peak area, b, showed good linear regression in the range from 0.75 to 125.00 mg/L with the equation a = 0.0189b + 1.1452 and R^2^ = 0.994.

### 3.2. Soil Sample Analysis

#### 3.2.1. Physico-Chemical Analysis

The chosen parameters were dynamic, scalable, simple to analyze and economical. These parameters were pH, electrical conductivity, organic matter, phosphorus, potassium and texture.

#### 3.2.2. ED-XRF Fluorescence Analysis

We performed an ED-XRF fluorescence analysis from the PANalytical Company to complete the physicochemical analysis of the soil. This technique allowed us to determine other important elements little known or unknown by farmers. These elements were SiO_2_, Al_2_O_3_, CaO, Fe_2_O_3_, K_2_O, MgO, MnO, Na_2_O, P_2_O_5_ and TiO_2_.

#### 3.2.3. X-ray Diffraction Analysis

The device used was an Empyrean diffractometer from PANalytical, equipped with a copper anticathode, *λ* k_α_ (Cu) = 1.5406, bombarded by electrons accelerated under a voltage of 45 kV, with a current of 35 mA.

The diffraction diagrams were recorded in continuous mode in an angular domain in 2θ ranging from 3° to 90° with a step of 0.066°.

### 3.3. Statistical Analysis

The statistical study concerned the variation of the main secondary metabolites of saffron according to the composition of soil. It was carried out using the ANOVA procedure of IBM SPSS Statistics 21 software. The multiple comparison of the means and their classification was carried out by Tukey’s test each time the analysis of variance revealed significant differences.

## 4. Conclusions

Environmental characteristics, particularly climate and soil, are among the factors that directly affect the production and quality of saffron secondary metabolites. In this work, we contributed to the study of the impact of soil on saffron secondary metabolites. Although saffron tolerates a wide range of biological conditions, some soils, with specific characteristics, perform better than others. We can conclude that the evaluation of soil conditions is particularly important to obtain high-quality saffron.

The soils T10, T2 and T1 produced saffron with the highest value of coloring strength, although 9 out of 10 saffron samples belonged to the first category. We found that saffron samples grown in loam–clay–sand soils contained high values of crocins and kaempferol 3-sophoroside 7-glucoside but low values of safranal. In addition, saffron samples grown in soils rich in organic matter, phosphorus and potassium contained high values of crocins and kaempferol 3-sophoroside 7-glucoside but low values of safranal.

Clays containing low amounts of iron could have a positive effect on the coloring strength of saffron. Furthermore, we observed that soil silicon had a positive effect on the arrangement of mineral elements and their distribution, particularly on the reduction of iron and manganese, thus limiting the risk of their toxicity. The potassium rate in the studied soils was related to the presence of muscovite and illite.

Based on the obtained results, we suggest improving the quality of soil deficient in the elements necessary for the *Crocus sativus* plant by amending it with clay rich in these elements; this would intensify the secondary metabolites of saffron and thus improve its quality.

## Figures and Tables

**Table 1:** Physicochemical results of the soil samples.

Samples	Sand (%)	Clay (%)	Loam (%)	Texture	pH	Class	EC (ms/cm)	Class	P_2_O_5_ (ppm)	Class	OM (%)	Class	K_2_O (ppm)	Class
T1	52.9	21.4	25.6	Sand–clay–loam	7.35	Weakly alkaline	3.72	Non-saline	225.99	Very high	6.18	Very rich	704.93	Very high
T2	45.9	20.8	33.3	Loam	7.1	Neutral	1.2	Non-saline	22.11	Low	3.6	Moderately rich	123.5	Rich
T3	64.5	15.4	20	Sand–loam	7.63	Weakly alkaline	0.9	Non-saline	13.77	Low	2.84	Moderately rich	69.29	Low
T4	47.2	21.2	31.5	Loam	7.87	Moderately alkaline	1.12	Non-saline	105.57	Very high	4.92	Rich	466.94	Very high
T5	47	15.7	37.3	Loam	7.48	Weakly alkaline	0.97	Non-saline	30.24	Rich	2.34	Moderately rich	189.79	High
T6	48	17.8	34.1	Loam	6.85	Neutral	2.08	Non-saline	95.85	High	7.34	Very rich	241	High
T7	49.2	15.4	35.4	Loam	7.49	Weakly alkaline	0.94	Non-saline	46.44	High	3.2	Rich	120.5	Rich
T8	47.4	27.2	25.4	Sand–clay–loam	7.5	Weakly alkaline	0.86	Non-saline	31.86	Rich	3.78	Rich	265.1	High
T9	62.8	15.4	21.8	Sand–loam	7.39	Weakly alkaline	1.2	Non-saline	0.27	Low	4.24	Rich	132.55	Rich
T10	52.5	21.3	26.2	Sand–clay–loam	7.38	Weakly alkaline	1.51	Non-saline	81.54	High	3.16	Rich	259.08	High

OM: organic matter, P_2_O_5_: phosphorus, K_2_O: potassium, EC: electrical conductivity.

**Table 2:** Effect of soil texture, pH, mineral elements and organic matter on the crocetin esters (g kg^−1^ of dry matter) of Moroccan saffron.

Texture	*Trans*-5-nG	*Trans*-4-GG	*Trans*-4-ng	*Trans*-3-Gg	*Trans*-2-gg	*Cis*-4-GG	*Trans*-2-G	*Cis*-3-Gg	*Cis*-2-G	*Trans*-1-g
Loam–clay–sand	3.90 ^b^ ± 0.58	103.76 ^b^ ± 7.81	3.56 ^b^ ± 0.84	78.46 ^c^ ± 9.19	14.20 ^b^ ± 3.91	2.62 ^b^ ± 0.67	1.00 ^a^ ± 0.26	10.62 ^b^ ± 1.62	0.39 ^b^ ± 0.08	3.42 ^b^ ± 1.25
Sand–loam	2.77 ^a^ ± 0.09	86.16 ^a^ ± 10.78	2.61 ^a^ ± 0.14	39.51 ^a^ ± 1.34	7.99 ^a^ ± 0.78	2.24 ^b^ ± 0.11	1.03 ^a^ ± 0.08	7.06 ^a^ ± 1.71	0.29 ^a^ ± 0.04	1.88 ^a^ ± 0.03
Loam	3.22 ^a^ ± 0.25	97.19 ^b^ ± 5.68	3.47 ^b^ ± 0.72	62.75 ^b^ ± 8.75	11.58 ^b^ ± 1.82	1.63 ^a^ ± 0.25	0.83 ^a^ ± 0.10	7.57 ^a^ ± 2.11	0.28 ^a^ ± 0.05	3.05 ^b^ ± 0.75
**pH**										
Weakly alkaline	3.42 ^a^ ± 0.66	97.21 ^a^ ± 10.71	3.21 ^a^ ± 0.82	64.77 ^a^ ± 17.50	11.92 ^a^ ± 3.95	2.23 ^a^ ± 0.68	0.96 ^a^ ± 0.21	8.86 ^a,b^ ± 2.42	0.35 ^b^ ± 0.08	2.92 ^a^ ± 1.15
Moderately alkaline	3.47 ^a^ ± 0.03	93.55 ^a^ ± 0.07	3.82 ^a^ ± 0.04	74.71 ^a^ ± 0.08	12.81 ^a^ ± 0.17	1.71 ^a^ ± 0.02	0.74 ^a^ ± 0.02	10.24 ^b^ ± 0.05	0.26 ^a.b^ ± 0.01	3.82 ^a^ ± 0.02
Neutral	3.19 ^a^ ± 0.03	104.88 ^a^ ± 0.03	3.84 ^a^ ± 0.04	51.05 ^a^ ± 0.02	10.95 ^a^ ± 0.03	1.89 ^a^ ± 0.01	0.95 ^a^ ± 0.02	5.76 ^a^ ± 0.01	0.22 ^a^ ± 0.01	2.47 ^a^ ± 0.02
**Phosphorus**										
Low	3.26 ^a^ ± 0.73	94.90 ^a^ ± 15.64	3.13 ^a^ ± 0.79	52.95 ^a^ ± 20.19	10.12 ^a^ ± 3.25	2.36 ^b^ ± 0.19	1.06 ^b^ ± 0.09	8.50 ^a,b^ ± 2.54	0.31 ^a^ ± 0.04	2.34 ^a^ ± 0.70
Rich	3.19 ^a^ ± 0.20	95.74 ^a^ ± 4.14	3.21 ^a^ ± 0.79	68.13 ^a^ ± 5.40	12.26 ^a^ ± 1.36	1.41 ^a^ ± 0.19	0.73 ^a^ ± 0.17	6.72 ^a^ ± 1.37	0.33 ^a^ ± 0.01	2.98 ^a^ ± 0.80
High	3.51 ^a^ ± 0.74	100.89 ^a^ ± 7.60	3.53 ^a^ ± 0.98	68.40 ^a^ ± 18.40	13.41 ^a^ ± 5.20	2.21 ^b^ ± 0.66	0.94 ^a,b^ ± 0.17	8.57 ^a,b^ ± 2.35	0.35 ^a^ ± 0.14	3.36 ^a^ ± 1.54
Very high	3.68 ^a^ ± 0.24	98.64 ^a^ ± 5.58	3.46 ^a^ ± 0.40	71.78 ^a^ ± 3.23	11.99 ^a^ ± 0.93	2.46 ^b^ ± 0.82	0.96 ^a,b^ ± 0.24	11.10 ^b^ ± 0.94	0.31 ^a^ ± 0.06	3.29 ^a^ ± 0.59
**Organic matter**										
Moderately rich	3.48 ^a^ ± 0.61	102.63 ^b^ ± 7.47	3.61 ^a^ ± 0.67	61.26 ^a^ ± 16.99	12.12 ^a^ ± 2.79	1.99 ^a^ ± 0.60	0.99 ^a^ ± 0.11	8.49 ^a^ ± 2.55	0.33 ^a^ ± 0.02	2.95 ^a^ ± 0.83
Rich	3.30 ^a^ ± 0.66	91.92 ^a^ ± 10.07	3.11 ^a^ ± 0.90	68.04 ^a^ ± 18.34	12.12 ^a^ ± 4.60	2.08 ^a^ ± 0.59	0.86 ^a^ ± 0.22	8.74 ^a^ ± 2.03	0.34 ^a^ ± 0.10	3.11 ^a^ ± 1.38
Very rich	3.54 ^a^ ± 0.39	104.30 ^b^ ± 0.64	3.47 ^a^ ± 0.41	59.95 ^a^ ± 9.76	11.05 ^a^ ± 0.21	2.55 ^a^ ± 0.72	1.07 ^a^ ± 0.13	8.86 ^a^ ± 3.40	0.29 ^a^ ± 0.08	2.61 ^a^ ± 0.16
**Potassium**										
Low	2.85 ^a^ ± 0.03	96.00 ^a^ ± 0.01	2.73 ^a^ ± 0.02	40.73 ^a^ ± 0.02	8.48 ^a^ ± 0.89	2.15 ^a^ ± 0.02	0.95 ^a^ ± 0.02	8.63 ^a,b^ ± 0.02	0.32 ^a^ ± 0.02	1.88 ^a^ ± 0.02
Rich	3.26 ^a^ ± 0.73	93.18 ^a^ ± 15.72	2.98 ^a^ ± 0.90	60.06 ^a,b^ ± 18.05	10.30 ^a,b^ ± 3.13	2.20 ^a^ ± 0.41	0.99 ^a^ ± 0.19	8.55 ^a,b^ ± 2.54	0.31 ^a^ ± 0.04	2.45 ^a,b^ ± 0.64
High	3.51 ^a^ ± 0.60	100.83 ^a^ ± 6.05	3.68 ^a^ ± 0.76	69.85 ^b^ ± 15.69	13.93 ^b^ ± 3.96	1.95 ^a^ ± 0.73	0.89 ^a^ ± 0.21	7.59 ^a^ ± 2.38	0.35 ^a^ ± 0.12	3.46 ^b^ ± 1.31
Very high	3.68 ^a^ ± 0.24	98.64 ^a^ ± 5.58	3.46 ^a^ ± 0.40	71.78 ^b^ ± 3.23	11.99 ^a,b^ ± 0.93	2.46 ^a^ ± 0.82	0.96 ^a^ ± 0.24	11.10 ^b^ ± 0.94	0.31 ^a^ ± 0.06	3.29 ^a,b^ ± 0.59

Values are averages. The different letters in superscript indicate significant differences according to Tukey’s test (*p* < 0.05).

**Table 3:** Effect of soil texture, pH, mineral elements and organic matter on coloring strength, kaempferol 3-sophoroside 7-glucoside, picrocrocin and safranal (g kg^−1^ of dry matter) of Moroccan saffron.

Texture	$E_{1\;cm}^{1\%}$ 440 nm	Kaempferol 3-Sophoroside 7-Glucoside	Picrocrocin	Safranal
Loam–clay–sand	253.63 ^c^ ± 23.15	11.29 ^b^ ± 0.93	134.33 ^a^ ± 30.57	2.20 ^a^ ± 1.33
Sand–loam	199.00 ^a^ ± 7.29	9.14 ^a^ ± 0.26	112.42 ^a^ ± 3.08	4.16 ^b^ ± 0.24
Loam	219.66 ^b^ ± 4.85	10.26 ^a,b^ ± 1.56	129.30 ^a^ ± 30.96	3.26 ^a,b^ ± 1.43
**pH**				
Weakly alkaline	230.81 ^a^ ± 29.24	10.25 ^a^ ± 1.40	129.39 ^a^ ± 30.73	2.95 ^a^ ± 1.49
Moderately alkaline	227.00 ^a^ ± 2.00	12.25 ^a^ ± 0.07	110.15 ^a^ ± 0.32	4.37 ^a^ ± 0.11
Neutral	217.66 ^a^ ± 0.58	10.24 ^a^ ± 0.09	134.08 ^a^ ± 0.10	2.19 ^a^ ± 0.11
**Phosphorus**				
Low	220.93 ^a^ ± 33.69	9.39 ^a^ ± 0.42	121.16 ^a^ ± 13.34	3.20 ^a^ ± 1.46
Rich	221.00 ^a^ ± 2.37	11.11 ^b^ ± 0.66	139.67 ^a^ ± 39.10	3.01 ^a^ ± 1.49
High	237.90 ^a^ ± 32.53	10.19 ^a,b^ ± 1.84	137.27 a ± 35.81	2.80 ^a^ ± 1.57
Very high	237.90 ^a^ ± 10.65	11.77 ^b^ ± 0.54	112.36 ^a^ ± 2.44	3.07 ^a^ ± 1.42
**Organic matter**				
Moderately rich	229.71 ^a^ ± 27.36	9.92 ^a^ ± 0.50	141.20 ^a^ ± 28.54	2.43 ^a,b^ ± 1.46
Rich	227.74 ^a^ ± 30.32	10.64 ^a^ ± 1.86	121.42 ^a^ ± 30.99	3.78 ^b^ ± 1.29
Very rich	231.66 ^a^ ± 15.58	10.77 ^a^ ± 0.58	124.33 ^a^ ± 10.68	1.98 ^a^ ± 0.24
**Potassium**				
Low	205.33 ^a^ ± 2.52	9.38 ^a^ ± 0.06	109.60 ^a^ ± 0.07	4.37 ^a^ ± 0.11
Rich	224.16 ^a^ ± 32.32	8.94 ^a^ ± 0.79	117.16 ^a,b^ ± 17.83	3.35 ^a^ ± 1.61
High	235.17 ^a^ ± 27.86	11.18 ^b^ ± 0.88	148.38 ^b^ ± 32.66	2.40 ^a^ ± 1.23
Very high	236.33 ^a^ ± 10.65	11.77 ^b^ ± 0.54	112.36 ^a,b^ ± 2.44	3.07 ^a^ ± 1.42

Values are averages. The different letters in superscript indicate significant differences according to Tukey’s test (*p* < 0.05).

**Table 4:** Mineral composition of soil samples determined by X-ray diffraction.

Minerals	Chemical Formula
Muscovite-1\ITM\RG, syn	KAl_2_Si_3_AlO_10_(OH)_2_
Muscovite 2\ITM\RG#1	KAl_3_Si_3_O_10_(OH)_2_
Quartz	SiO_2_
Albite (ordered, high)	NaAlSi_3_O_8_ K_0.2_Na_0.8_AlSi_3_O_8_
Illite-2\ITM\RG#1 [NR]	(K, H_3_O)Al_2_Si_3_AlO_10_(OH)_2_
Albite, calcian, ordered	(Na, Ca)Al(Si, Al)_3_O_8_ (Na, Ca) (Si, Al)_4_O_8_
Aerinite	[(Fe^+2^, Fe^+3^, Al )_3_Mg_3_(Ca, Na)_4_[Si1_3.5_Al_4.5_O_42_] (OH)_6_]_11.3_ H_2_O

**Table 5:** Mineral elements of soil samples determined by ED-XRF fluorescence and X-ray diffraction.

	SiO_2_ (%)		Al_2_O_3_ (%)		CaO (%)		Fe_2_O_3_ (%)		K_2_O (%)		MgO (%)		MnO (%)		Na_2_O (%)		P_2_O_5_ (%)		TiO_2_ (%)	
Samples	XF	XDR	XF	XDR	XF	XDR	XF	XDR	XF	XDR	XF	XDR	XF	XDR	XF	XDR	XF	XDR	XF	XDR
T1	55.00	49.50	16.30	18.80	3.20	3.92	9.20	2.80	2.40	3.00	2.90	2.80	0.20	0.00	1.20	8.20	0.10	0.00	1.20	0.00
T2	52.40	43.60	16.40	16.70	2.20	3.78	8.40	2.70	2.70	2.80	2.20	2.70	0.20	0.00	1.20	6.70	0.20	0.00	1.10	0.00
T3	52.20	49.70	15.30	18.20	3.10	4.90	9.30	3.50	2.70	2.40	3.10	3.50	0.20	0.00	1.10	8.90	0.20	0.00	1.20	0.00
T4	54.30	33.20	15.70	28.10	2.30	5.10	7.50	0.00	3.00	5.70	2.40	0.00	0.20	0.00	0.90	2.70	0.00	0.00	1.00	0.00
T5	51.00	40.10	15.20	19.00	2.40	4.20	9.00	3.00	2.00	3.10	2.40	3.00	0.10	0.00	1.40	7.70	0.10	0.00	1.20	0.00
T6	53.80	40.60	13.90	12.60	3.10	5.60	9.70	4.00	2.60	0.00	3.10	4.00	0.20	0.00	0.50	10.20	0.20	0.00	1.30	0.00
T7	54.40	43.30	14.70	28.00	3.00	9.60	9.50	3.00	2.30	4.20	2.80	3.00	0.20	0.00	1.40	5.00	0.20	0.00	1.30	0.00
T8	53.10	23.40	16.40	10.00	3.10	0.00	8.80	0.00	2.50	2.20	2.40	0.00	0.20	0.00	0.70	3.40	0.10	0.00	1.20	0.00
T9	51.60	46.20	15.20	17.90	2.80	4.20	9.60	3.00	2.40	2.80	2.90	3.00	0.20	0.00	0.50	7.30	0.10	0.00	1.20	0.00
T10	60.70	47.30	16.20	29.20	1.80	10.20	7.10	4.10	3.10	4.80	2.00	4.10	0.20	0.00	2.00	5.30	0.00	0.00	0.90	0.00

XF: ED-XRF fluorescence. XDR: X-ray diffraction.

## Data Availability

Not applicable.

## Appendix A

Triangular diagram of textural classes of the studied soils.

## Appendix B

Diffractogram of X-rays on powder and on oriented blades of the T1 sample.
